# Granulocytic Myeloid-Derived Suppressor Cells (GR-MDSC) in Breast Milk (BM); GR-MDSC Accumulate in Human BM and Modulate T-Cell and Monocyte Function

**DOI:** 10.3389/fimmu.2018.01098

**Published:** 2018-05-17

**Authors:** Natascha Köstlin, Carolin Schoetensack, Julian Schwarz, Bärbel Spring, Alexander Marmé, Rangmar Goelz, Gerhard Brodbeck, Christian F. Poets, Christian Gille

**Affiliations:** ^1^Department of Neonatology, Tuebingen University Children’s Hospital, Tuebingen, Germany; ^2^Private Practice in Gynecology and Obstetrics, Tuebingen, Germany; ^3^Department of Hematology, Tuebingen University Children’s Hospital, Tuebingen, Germany

**Keywords:** myeloid-derived suppressor cell, breast milk, TLR4, oxytocin, prolactin

## Abstract

Nosocomial bacterial infections (NBI) and necrotizing enterocolitis (NEC) are among the main reasons for death in preterm infants. Both are often caused by bacteria coming from the infected infant’s gut and feeding with breast milk (BM) seems beneficial in their pathogenesis. However, mechanisms causing the protective effect of BM are only incompletely understood. Myeloid-derived suppressor cells (MDSC) are myeloid cells with suppressive activity on other immune cells, recently described to play a role in mediating maternal–fetal tolerance during pregnancy and immune adaptation in newborns. Until now, nothing is known about occurrence and function of MDSC in BM. We analyzed MDSC in BM and peripheral blood of breastfeeding mothers and found that granulocytic MDSC, but not monocytic MDSC, accumulate in BM, exhibit an activated phenotype and increased suppressive activity and modulate TLR-expression on monocytes. Furthermore, we found that the lactotrophic hormones prolactin and oxytocin do not induce MDSC from peripheral blood. This is the first study to describe MDSC with immune-modulatory properties in human BM. Our results point toward a role for MDSC in local immune modulation in the gut possibly protecting infants from NBI and NEC.

## Introduction

Preterm delivery is the main reason for perinatal morbidity and mortality (1). Besides respiratory complications, bacterial infections and necrotizing enterocolitits (NEC) are the most important causes of death in preterm infants (2). Bacterial infections in preterm infants mainly occur as nosocomial bacterial infections (NBI), compromising up to one of three very low birth weight infants (VLBW) (3). Evidence exists that pathogens causing NBI in preterm infants often descend from the intestinal flora of affected infants (4). Breast milk (BM) feeding prevents NBI (5, 6). NEC affects about 7% of all VLBW infants with a mortality rate of 20–30% (7). Besides preterm delivery, one of the main risk factors for NEC is formula instead of BM feeding (7–10). However, until now, mechanisms by which BM prevents NBI and NEC are incompletely understood.

Myeloid derived suppressor cells (MDSC) are myeloid cells with suppressive activity on innate and adaptive immune cells primarily described in cancer patients suppressing immune response against the tumor (11). On basis of their surface markers, MDSC can be subdivided in granulocytic MDSC (GR-MDSC) expressing granulocytic markers like CD66b and/or CD15 and monocytic MDSC (MO-MDSC) expressing the monocytic antigen CD14 (12). Differentiation of GR-MDSC from mature granulocytes in peripheral blood is—besides demonstrating their suppressive activity—mainly possible by separation according to their density (13), since GR-MDSC sediment with the low density fraction. The main mechanisms by which MDSC exert their suppressive activity on other immune cells are the depletion of arginine and tryptophan by expression of effector enzymes arginase I (ArgI), inducible NO-synthase (iNOS), and indolamin-2,3-dioxygenase (IDO), as well as the production of reactive oxygen species (ROS) (11). Recently, we could show that GR-MDSC, but not MO-MDSC accumulate during pregnancy in the maternal and fetal organism contributing to maternal–fetal tolerance on the one hand and to postnatal immune adaptation in neonates on the other (14–19). Although it is well known that BM contains lots of immune cells (20, 21), nothing is known about the occurrence and function of MDSC in human BM.

In the present study, we analyzed expression of MDSC in BM in comparison to peripheral blood of breastfeeding mothers and show that (1) GR-MDSC accumulate in BM, (2) GR-MDSC from BM (BM-MDSC) strongly suppress T-cell proliferation, (3) BM-MDSC exhibit an activated phenotype in comparison to GR-MDSC from peripheral blood with altered expression of chemokine receptors, co-inhibitory molecules, and effector enzymes and higher suppressive capacity, (4) BM-MDSC modulate toll-like receptor (TLR)-expression of monocytes, and (5) MDSC are not induced by lactotrophic hormones oxytocin and prolactin. These results point toward a novel role of GR-MDSC in BM for regulation of intestinal immune responses in neonates. Targeting BM-MDSC might be a strategy to prevent severe complications like NBI and NEC in preterm infants.

## Materials and Methods

### Patients

The local ethics committee approved this study (682/2016BO1) and all women gave their written informed consent. From November 2016 to November 2017, BM and peripheral blood from breastfeeding mothers of healthy term infants were collected during routine blood sampling 6 weeks postpartum. BM from breastfeeding mothers of preterm infants was collected during their stay in the Department of Neonatology at Tuebingen University Hospital starting in the first week of life and then weekly for 6 weeks. Women with chronic or acute infections at the time of sample collection were excluded from the study.

### Cell Isolation and Flow Cytometry

All samples were processed within 6 h after collection. Peripheral blood mononuclear cells (PBMC) were prepared from EDTA blood samples for phenotype analyses and from heparinized blood samples (4 IE/ml) for functional analyses by Ficoll density gradient centrifugation (lymphocyte separation medium, Biochrom, Berlin, Germany).

For isolation of milk cells, BM was diluted in a 1:1 ratio with phosphate buffered saline (PBS) and centrifuged for 20 min at 805 *g*. Supernatant was removed and cells were washed two times with PBS (22).

For GR-MDSC isolation from PBMC or milk cells, cells were labeled with anti-CD66b-FITC, followed by two sequential anti-FITC magnetic bead separation steps (MiltenyiBiotec, Bergisch-Gladbach, Germany) according to the manufacturer’s instructions. Purity of CD66b^+^ cells after separation was >90%, as assessed by flow cytometry.

After Ficoll density gradient centrifugation GR-MDSC were characterized as CD66b^+^/CD33^+^/CD14^−^/HLA-DR^low/−^ cells and MO-MDSC as CD14^+^/HLA-DR^low/−^, according to previously established human MDSC characterization methods (16). Antibody against ILT4 was purchased from R&D Systems, Wiesbaden-Nordenstadt, Germany. All other antibodies for extracellular staining were purchased from BD Pharmingen, Heidelberg, Germany.

Intracellular staining for MDSC effector enzymes ArgI, iNOS, and IDO was performed as described previously (15). Antibody against ArgI and IDO were purchased from R&D systems, Wiesbaden-Nordenstadt, Germany, antibody against iNOS from Santa Cruz, Heidelberg, Germany.

Data acquisition was performed with a FACScalibur flow cytometer (BD Bioscience) and analyzed *via* CellQuest (BD Biosciences).

### *In Vitro* Digestion Model

For *in vitro* digestion of BM, we used a previously established protocol by Klitgaard et al. (23). In brief, 25 ml of BM were added to concentrated gastric medium leading to a final concentration of 3 mM NaCl, 2 mM TRIS, and 2 mM maleic acid. pH was adjusted to 6.4 by adding 2.5% HCl. Then, gastric lipase (17 TBU/ml) and pepsin (126 U/ml) were added and mixture was incubated for 50 min at 37°C. pH was kept constant (6.1–6.5) by addition of 0.5 M NaOH. After 50 min, 11 ml of concentrated intestinal medium was added leading to a final concentration of 89.5 mM NaCl, 2 mM TRIS, 2 mM maleic acid, 1 mM sodiumtaurocholate, 0.2 mM phospholipids. pH was adjusted to 6.5 by adding 2.5% HCl. Then, pancreatin (50 TBU/ml pancreatic lipase) was added and mixture was incubated for another 90 min at 37°C. pH was kept constant (6.1–6.6) by addition of 0.5 M NaOH. Afterward, milk cells were isolated as described above.

### Cytospins

Isolated BM-MDSCs were fixed with cytospin centrifugation and stained with May–Gruenwald–Giemsa. Images were acquired on a Carl Zeiss Fotomicroscope (40× Planapo oil objective, Carl Zeiss, Oberkochen, Germany) using a Canon EOS 500 camera (Canon, Krefeld, Germany) and Adobe Photoshop CS3 software (Adobe Systems, Dublin, Ireland).

### T-Cell Suppression Assays

Peripheral blood mononuclear cell from healthy, non-pregnant adults were isolated, stained with carboxyfluorescein-succinimidyl ester (CFSE, Invitrogen, Heidelberg, Germany) according to the manufacturer’s instructions and stimulated with 100 U/ml interleukin-2 (IL-2, R&D Systems, Wiesbaden-Nordenstadt, Germany) and 1 µg/ml OKT3 (Janssen-Cilag, Neuss, Germany). 60,000 PBMC per well in RPMI1640 supplemented with 10% autologous serum were seeded in a 96-well microtiter plate (BD Biosciences) and 20,000, 30,000, or 60,000 GR-MDSC in RPMI1640, isolated from PBMC of breastfeeding women or from milk cells were added. As control, only RPMI1640 was added to the PBMC. After 96 h incubation, cells were harvested and stained with anti-CD8-PE and anti-CD4-APC (BD Pharmingen). CFSE fluorescence intensity was analyzed by flow cytometry to determine proliferation of CD4^+^ and CD8^+^ T-cells. Proliferation index, defined as the ratio of T-cell proliferation after addition of GR-MDSC and T-cell proliferation without GR-MDSC, was determined. T-cell proliferation without GR-MDSC was set to a fixed value of 1.

### ROS Detection

For detection of ROS, 4 × 10^5^ PBMC or milk cells were incubated with dihydrorhodamine 123 (DHR, Sigma, Munich, Germany) in RPMI1640 for 5 min at 37°C. Thereafter, cells were stimulated for 10 min with 60 ng/ml of Phorbol-12 myristate-13 acetate (PMA, Sigma, Munich, Germany). Cells were washed, surface stained with anti-CD66b-APC (eBiosciences, San Diego, CA, USA) and ROS production was analyzed by flow cytometry.

### Coculture Experiments

Peripheral blood mononuclear cell from healthy, non-pregnant individuals were isolated and seeded at a concentration of 1 × 10^6^ cells/ml in RPMI1640 with 10% fetal calf serum (FCS) in a 24-well plate and 2.5 × 10^5^ BM-MDSC were added. As control, only RPMI1640 was added to the PBMC. After 5 days of culture surface staining for CD66b, CD14, TLR2, and TLR4 (all from BD biosciences) was performed.

### MDSC Induction

Human PBMC were isolated from heparinised blood samples (4 IE/ml) from healthy volunteer donors by Ficoll density gradient centrifugation and cultured in complete medium (Dulbecco´s modified eagle medium) (Thermo Fisher Scientific, Darmstadt, Germany), supplemented with 10% FCS (Biochrom, Berlin, Germany), and 1% penicillin/streptomycin (Biochrom, Berlin, Germany) (5 × 10^5^ cells/ml) in 12-well-plates (Greiner Bio-One GmbH, Frickenhausen, Germany) supplemented with different concentrations of prolactin (Merck KGaA, Darmstadt, Germany) and oxytocin (Hexal AG, Holzkirchen, Germany). PBMC cultured in medium alone were run in parallel as induction negative control and PBMC cultured in medium with 1 ng/ml GM-CSF (R&D systems) as induction positive control (24). After 6 days, PBMC were removed using the non-protease cell detachment solution Detachin (GenLantis, San Diego, CA, USA). Since CD66b is downregulated after several days of culture, we quantified and enriched MDSC using the myeloid marker CD33^+^ according to previously described protocols (24, 25). CD33^+^ cells were quantified by flow cytometry.

### Statistical Analysis

Statistical analysis was done using GraphPad Prism 5.0 (GraphPad Software, La Jolla, CA, USA). Data were analyzed for Gaussian distribution using D’Agostino and Pearson omnibus normality test. Normally distributed data were analyzed using the paired *t*-test, not normally distributed data were evaluated using the Wilcoxon matched pairs signed rank test. A *p*-value < 0.05 was considered as statistically significant.

## Results

### GR-MDSC Accumulate in Human BM

**Figure 1:**
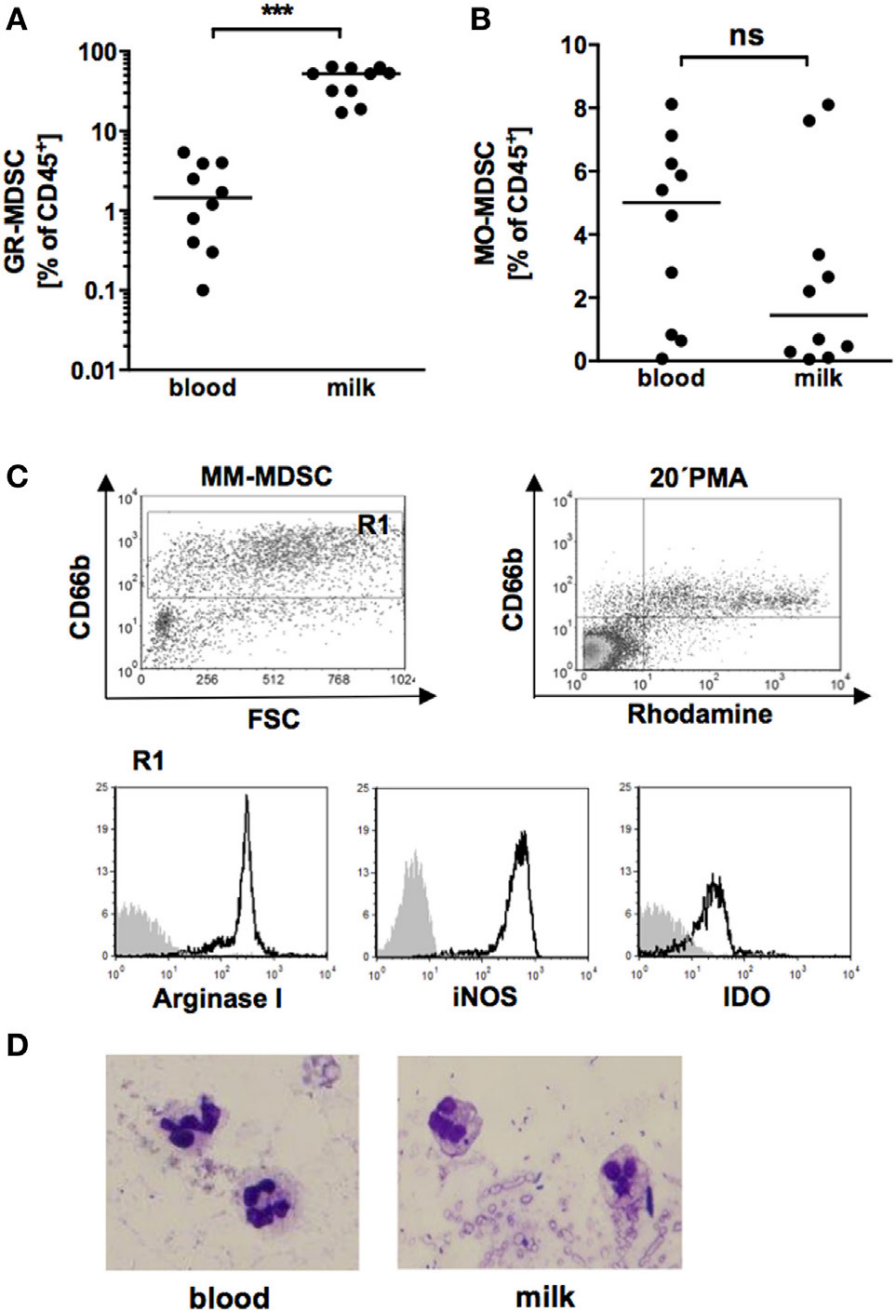
Quantification of granulocytic MDSC (GR-MDSC) and monocytic MDSC (MO-MDSC) in peripheral blood and breast milk (BM) of breastfeeding women. Mononuclear cells were isolated from peripheral blood of breastfeeding women and milk cells isolated from corresponding BM samples and analyzed by flow cytometry. **(A,B)** Scatter diagrams showing percentages of GR-MDSC **(A)** and MO-MDSC **(B)** from peripheral blood (blood) and BM (milk). Plots represent 10 independent experiments. Each symbol represents an individual sample and the median is indicated. ***p < 0.005; Wilcoxon matched pairs signed rank test. **(C)** Density plot of forward scatter vs. CD66b and Rhodamine vs. CD66b from CD45^+^ milk leukocytes. Gate 1 shows the GR-MDSC population. Representative histograms show the expression of intracellular effector enzymes ArgI, inducible NO synthase (iNOS) and IDO on GR-MDSC from BM. **(D)** Representative cytospin of GR-MDSC enriched from milk cells by MACS.

First, we analyzed expression of GR-MDSC in BM of term infants in comparison to peripheral blood of the corresponding mother. We quantified MDSC in total milk cells without previous isolation of mononuclear cells, as density gradient centrifugation of single samples showed that all milk cells sedimented with the low density fraction of mononuclear cells. We found that percentages of GR-MDSC were about 20-fold higher in BM than in peripheral blood (median 52.2% vs. 1.5%, *p* = 0.001, *n* = 10, Figure 1A), while percentages of MO-MDSC tended to be lower in BM (median 1.4 vs. 5.0%, *p* = 0.05, *n* = 10, Figure 1B). Figure S1 in Supplementary Material shows surface phenotype of BM-MDSC and GR-MDSC from peripheral blood. As shown in Figure 1C, BM-MDSC expressed the known effector enzymes ArgI, iNOS, and IDO and produced significant amounts of ROS. BM-MDSC showed the typical morphology of granulocytic cells with segmented nucleus (Figure 1D). Repeated measurements of BM from four preterm infants demonstrated that GR-MDSC counts did not decrease during the first seven postnatal weeks (Figure S2 in Supplementary Material). *In vitro* digestion with gastric and pancreatic enzymes showed that cells survived the gastric and intestinal passage; however, percentages of BM-MDSC of total leukocytes decreased after digestion as did the MFI of CD66b on BM-MDSC (Figure S3 in Supplementary Material).

### BM-MDSC Efficiently Suppress T-Cell Proliferation

**Figure 2:**
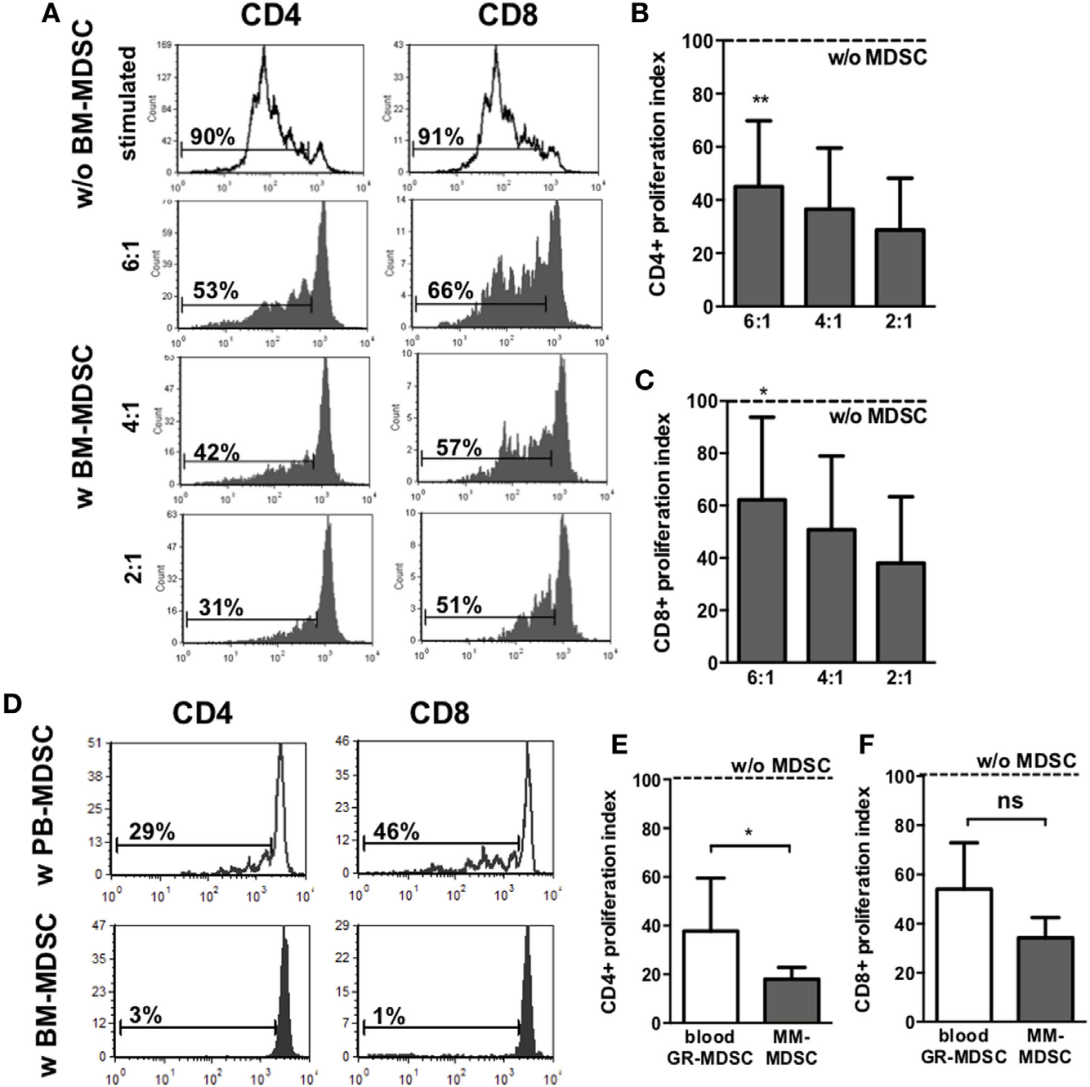
Inhibiton of CD4^+^ and CD8^+^ T-cell proliferation by granulocytic MDSC (GR-MDSC) from breast milk (BM). GR-MDSC were enriched from BM (BM-MDSC) and added to carboxyfluorescein-succinimidyl ester (CFSE)-stained and IL-2/OKT3-stimulated peripheral blood mononuclear cell (PBMC) isolated from a healthy adult control. After 4 days, proliferation of CD4^+^ and CD8^+^ T-cells was assessed by CFSE dye dilution. Proliferation index was determined as ratio of T-cell proliferation with and without addition of BM-MDSC. **(A)** Representative histogram plots showing proliferation of CD4^+^ T-cells and CD8^+^ T-cells with (gray histograms) and without (white histograms) addition of BM-MDSC in different proportions (ratio PBMC:BM-MDSC 6:1, 4:1, and 2:1). **(B,C)** Inhibitory effect of BM-MDSC on proliferation of CD4^+^ **(B)** and CD8^+^ T-cells **(C)**. Dashed line shows proliferation of target PBMC without addition of BM-MDSC. Inhibition of T-cell proliferation by BM-MDSC was measured at the indicated ratios by CFSE dye dilution. Bars show mean and SD of seven samples pooled from seven independent experiments. *p < 0.05, **p < 0.01 compared with target cells alone; Wilcoxon matched-pairs signed-rank test. **(D)** Representative histogram plots (from five independent experiments) showing proliferation of CD4^+^ T-cells and CD8^+^ T-cells after stimulation with OKT3 and IL-2 and addition of GR-MDSC isolated from PBMC from peripheral blood (white histograms) and BM-MDSC of the same person (gray histograms) in a 2:1 ratio. **(E,F)** The inhibitory effect of GR-MDSC from peripheral blood (blood GR-MDSC, white bars) and from BM (BM-MDSC, gray bars) on proliferation of CD4^+^ **(E)** and CD8^+^ T-cells **(F)** were assessed. Dashed line shows proliferation of target PBMC without addition of GR-MDSC. Inhibition of T-cell proliferation by GR-MDSC was measured at the indicated ratios by CFSE dye dilution. Bars show mean and SD of five independent experiments. *p < 0.05; ns not significant; Wilcoxon matched-pairs signed-rank test.

Next, we aimed to confirm that the granulocytic cells we found in BM and hypothesized to be MDSC indeed possess suppressive activity, the main characteristic to distinguish them from mature granulocytes. Therefore, we performed T-cell-proliferation assays with enriched BM-MDSC and PBMC from healthy adult donors. Figure 2A shows representative histogram plots for proliferation of adult and neonatal CD4^+^ and CD8^+^ T-cells with and without addition of BM-MDSC. Addition of BM-MDSC to PBMC reduced proliferation index of CD4^+^ T-cells concentration-dependent to 45.0 (1:6), 36.6 (1:4), and 28.7% (1:2) (*p* < 0.01 for 1:6, *n* = 7, Figure 2B). Accordingly, CD8^+^ T-cell-proliferation was reduced concentration-dependent to 62.2 (1:6), 50.8 (1:4), and 38.0% (1:2) (*p* < 0.05 for 1:6, *n* = 7, Figure 2C). Similar results were obtained when cord blood mononuclear cell were used as target cells (Figure S4 in Supplementary Material). Comparison of suppressive capacity of BM-MDSC and MDSC from peripheral blood revealed a stronger activity of BM-MDSC (proliferation index 17 vs. 38% for inhibition of CD4^+^ T-cell proliferation and 33 vs. 58% for inhibition of CD8^+^ T-cell proliferation, *n* = 5, *p* < 0.05 for CD4, not significant for CD8) (Figures 2D–F).

### BM-MDSC Express Increased Levels of Chemokine Receptors, Co-Inhibitory Molecules, and Effector Enzymes

**Figure 3:**
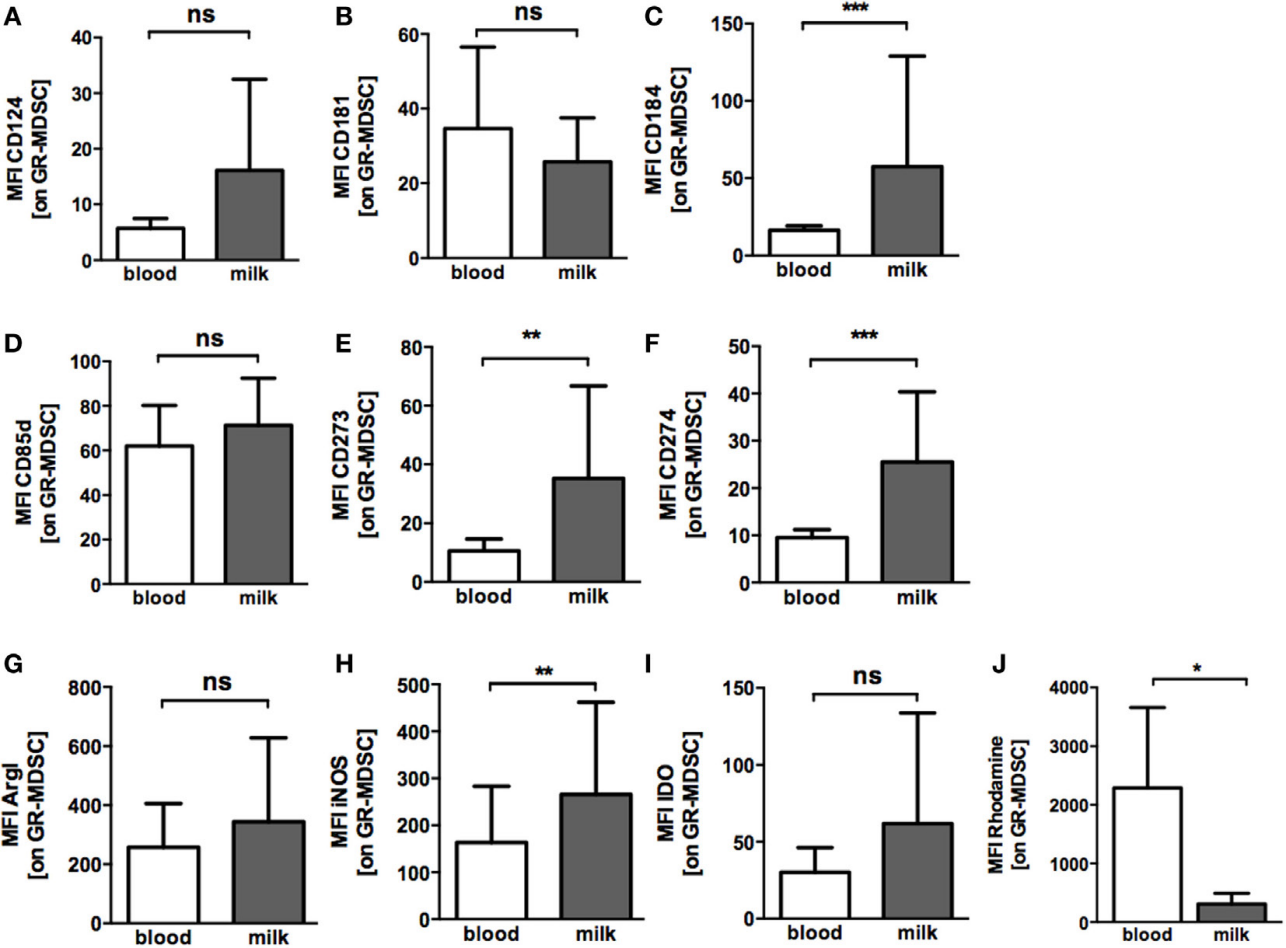
Expression of chemokine receptors, co-inhibitory molecules and effector enzymes on granulocytic MDSC (GR-MDSC) from BM. Mononuclear cells were isolated from peripheral blood of breastfeeding women and milk cells isolated from BM of the same person and analyzed by flow cytometry. Intracellular staining for arginase I (ArgI), iNOS, and IDO was performed and reactive oxygen species (ROS)-production detected by dihydrorhodamine. **(A–J)** Bar graphs show the MFI for receptor and enzyme expression on GR-MDSC in peripheral blood (blood, white bars) and BM (milk, gray bars). Bars represent data from 5 to 10 independent experiments and mean and SD are indicated. *p < 0.05; **p < 0.01; ***p < 0.005; ns, not significant; Wilcoxon matched-pairs signed rank test or paired *t*-test.

To get further insights into the activation state of BM-MDSC, we next asked whether BM-MDSC express different levels of chemokine receptors, activation markers, co-inhibitory molecules and effector enzymes than GR-MDSC from peripheral blood. BM-MDSC expressed significantly higher levels of CXCR4 (CD184) than PB-MDSC (MFI median 34.0 vs. 16.3, *p* = 0.001, *n* = 10 Figure 3C), while expression of CXCR1 (CD181) and IL4Rα (CD124) was not different (Figures 3A,B). Expression of the inhibitory receptor ILT4 (CD85d), that was recently shown to be relevant for activation of GR-MDSC during pregnancy, also did not differ between BM-MDSC and PB-MDSC (Figure 3D). However, BM-MDSC expressed higher levels of co-inhibitory molecules PD-L1 (CD274) and PD-L2 (CD273) than PB-MDSC (MFI median 20.0 vs. 9.0 for PD-L1 and 26.5 vs. 10.0 for PD-L2, *p* = 0.001 and *p* = 0.002, *n* = 10, Figures 3E,F). Regarding effector mechanisms, we found an increased expression of iNOS (MFI median 192.9 vs. 134.0, *p* = 0.006, *n* = 9, Figure 3H) and a decreased production of ROS (MFI median 343.0 vs. 2873.0, *p* = 0.03, *n* = 5, Figure 3J) by BM-MDSC, while expression of ArgI and IDO was similar in BM-MDSC and PB-MDSC (Figures 3G,I).

### BM-MDSC Modulate TLR-Expression on Monocytes

**Figure 4:**
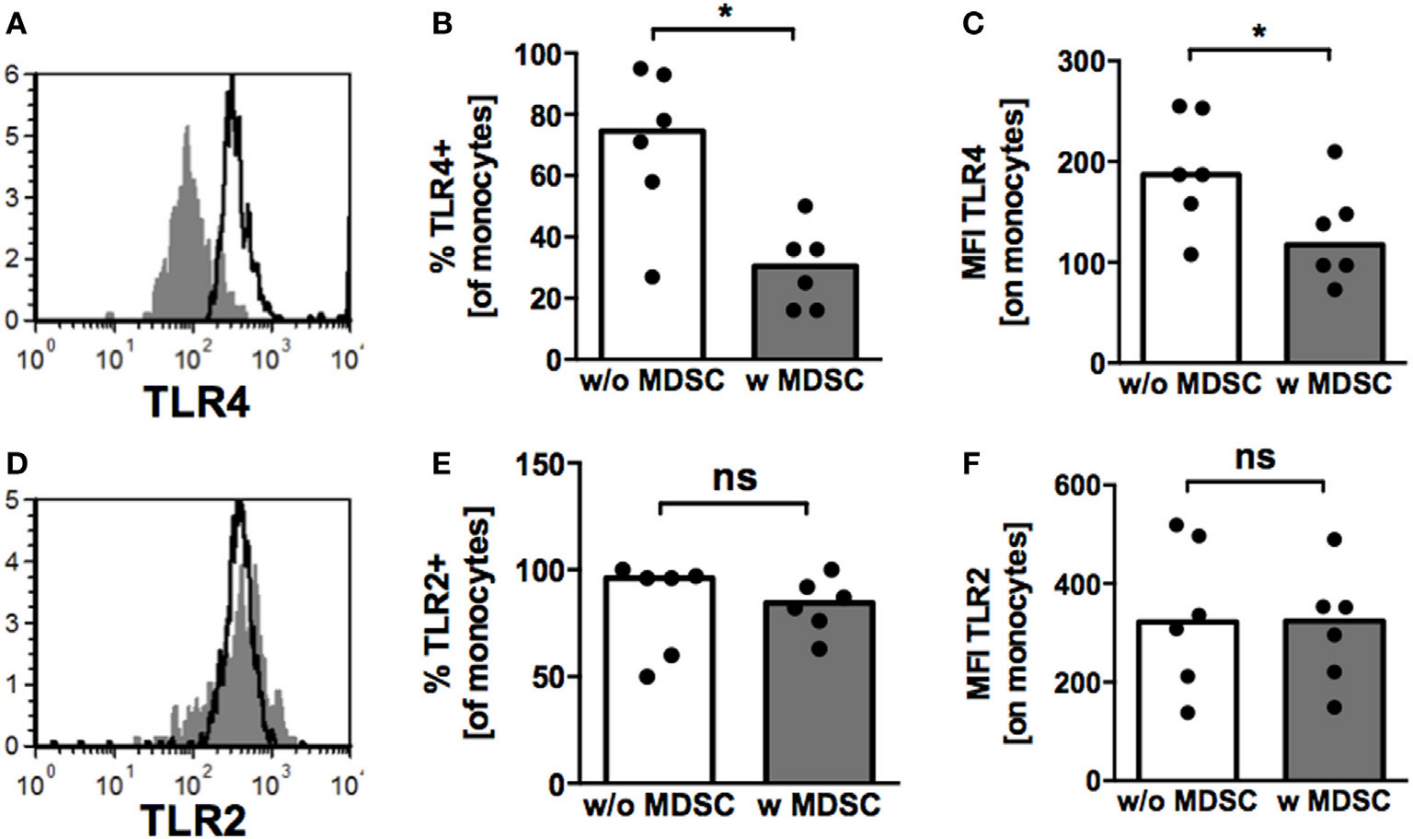
Toll-like receptor (TLR)-expression on monocytes under the influence of granulocytic MDSC (GR-MDSC) from breast milk (BM). GR-MDSC were enriched from BM and added to peripheral blood mononuclear cell isolated from a healthy adult control. After 5 days of culture, surface staining for TLR2 and TLR4 was performed and cells were analyzed by flow cytometry. **(A,D)** Representative histograms of TLR4- **(A)** and TLR2- **(D)** expression with (gray histograms) and without (white histograms) addition of GR-MDSC from BM. **(B,E)** Bar graphs showing the percentage of TLR4- **(B)** and TLR2- **(E)** expressing monocytes with (gray bars) and without (white bars) addition of BM-MDSCs. **(C,F)** Bar graphs showing the MFI of TLR4- **(C)** and TLR2- **(F)** expression on monocytes without addition of BM-MDSCs (white bars) and with addition of BM-MDSCs (gray bars). Bars represent pooled data from six independent experiments and each point represents an individual sample. *p < 0.05, ns not significant; Wilcoxon matched pairs signed rank test.

Expression of TLRs on monocytes plays an important role in bacterial host defense and pathogenesis of NEC (26, 27). Therefore, we examined TLR2 and TLR4-expression on monocytes after coculture with BM-MDSC. Both, percentages of TLR4-expressing cells (median 30.5 vs. 74.5%, *p* = 0.02, *n* = 6) (Figure 4B) and TLR4-MFI (127.2 ± 49.3 vs. 191.3 ± 56.5, *p* < 0.05, *n* = 6) (Figures 4A,C) were reduced on monocytes after coculture with BM-MDSC, while expression of TLR2 remained unchanged (Figures 4D–F).

### Prolactin and Oxytocin Do Not Induce MDSC From PBMC

**Figure 5:**
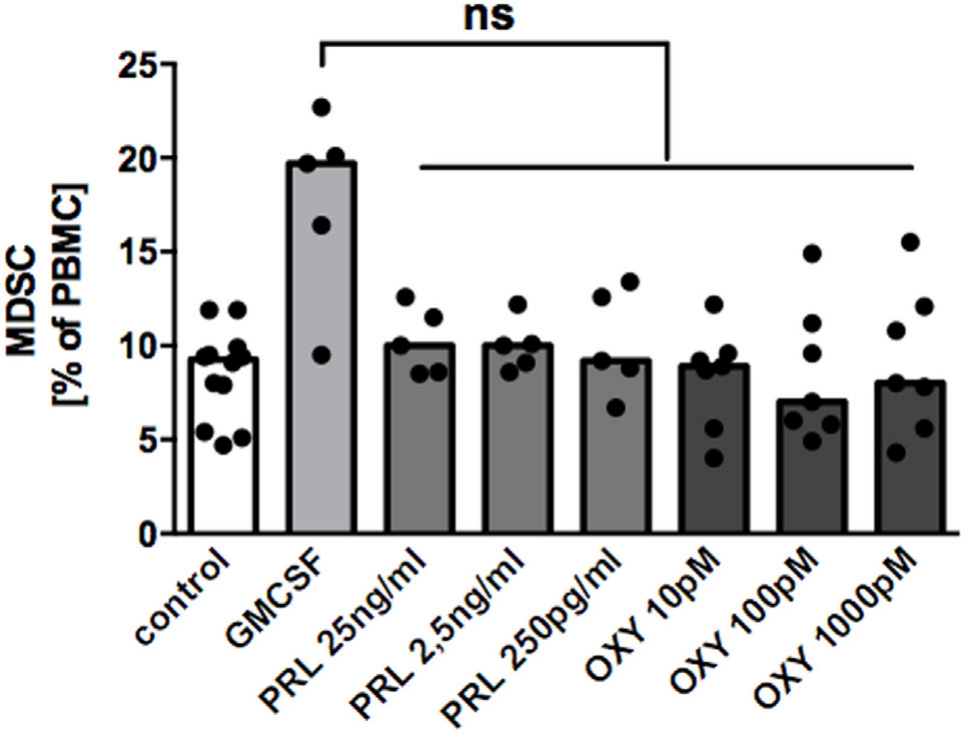
Effect of oxytocin and prolactin on induction of MDSC from peripheral blood mononuclear cell (PBMC). PBMC from healthy non-pregnant donors were cultured for 6 days either in medium only, in medium with 1 ng/ml GM-CSF, or in medium with oxytocin (OXY) or prolactin (PRL) in indicated concentrations. After 6 days, cells were harvested and percentages of CD33^+^ cells analyzed by flow cytometry. Bar graph shows percentages of CD33^+^ MDSC after culture in medium alone (white bar), in medium with 1 ng/ml GM-CSF (light gray bar), or with oxytocin (OXY, medium gray bar) or prolactin (PRL, dark gray bar) in indicated concentrations. Bars represent pooled data from five to seven experiments and each point represents an individual sample. ns, not significant; Friedmann test and Dunn’s multiple comparison test.

With intent to identify mechanisms by which MDSC-accumulation in BM occurs, we tested the hypothesis that the lactation-associated hormones oxytocin and/or prolactin could induce MDSC from PBMC according to previously described protocols (17, 24). However, neither prolactin nor oxytocin in different concentrations could induce MDSC from PBMC (Figure 5).

## Discussion

Expression of MDSC under pathological conditions has been widely described (13, 28–31). Beyond that, an accumulation of MDSC during healthy pregnancy both in maternal and fetal organism has been reported (14–19, 32–34) and a role for maternal–fetal tolerance and postnatal immune adaptation in neonates been postulated. Although it is well known that breastfeeding strongly contributes to establishment of neonatal immune defense, nothing was known on the presence and function of MDSC in BM. In the present study, we analyzed occurrence of MDSC in human BM and found that (1) GR-MDSC, but not MO-MDSC, accumulate in BM, (2) BM-MDSC efficiently suppress T-cell proliferation and are activated in comparison to MDSC from peripheral blood, (3) BM-MDSC exhibit an altered phenotype in comparison to peripheral blood MDSC of pregnant women, (4) BM-MDSC reduce TLR4-expression on monocytes, and (5) MDSC are not induced by lactotrophic hormones prolactin and oxytocin.

Our finding that GR-MDSC, but not MO-MDSC, accumulate in human BM is in line with results in peripheral blood of pregnant women and in cord blood of healthy newborns (14, 15), where we found increased levels of GR-MDSC, while numbers of MO-MDSC were unchanged when compared with peripheral blood of healthy adults. Recent *in vitro* and *in vivo* data revealed that systemic accumulation of GR-MDSC during pregnancy contributes to maternal–fetal tolerance and maintenance of pregnancy (33, 35), while they modulate adaptive immunity postnatally, potentially increasing the neonate’s susceptibility to infections (18, 19). The present study is the first describing the presence of MDSC also in BM. Contrary to our findings in peripheral blood of preterm infants (19), numbers of GR-MDSC in BM did not decrease during the first six postnatal weeks. Transfer of BM-MDSC from mother to child beyond the neonatal period could be a mechanism for local immune modulation in the gut, where the main contact with microbes takes place.

One important limitation of our study is that, due to high cell loss, quantification of MDSC in BM was performed without prior density gradient centrifugation. Since GR-MDSC and mature granulocytes exhibit the same surface phenotype and only differ by their density (13) some of the cells quantified as GR-MDSC in this study might have been granulocytes. Density gradient centrifugation of single milk samples, however, showed that all CD66b^+^ cells sedimented with mononuclear cells at the interphase and did not pellet with polymorph nuclear cells, supporting our assumption that CD66^+^ cells in BM are GR-MDSC. Much more important, however, is the fact that functional analyses of enriched milk CD66^+^ cells revealed a high T-cell suppressive activity of these cells—the key distinguishing feature between GR-MDSC and granulocytes. Suppressive activity of BM-MDSC was even stronger than that of blood MDSC. Previous studies quantifying leukocyte subpopulations in BM found similar percentages of neutrophilic cells as we did (21, 36). Trend et al. analyzed total milk cells and used a gating strategy to differentiate leukocyte subpopulations (21) with CD16 as marker for mature neutrophils which can be also expressed by GR-MDSC (13, 15). As they did not analyze the suppressive activity of these cells it remains unclear whether the cells they quantified were GR-MDSC or mature neutrophils. Maschmann et al. analyzed mononuclear cells after density gradient centrifugation and used CD66b as surface marker making it likely that the cells they named neutrophils were in fact GR-MDSC (36). Based on our functional findings, we now suppose that most neutrophilic cells in BM are GR-MDSC.

Another limitation of our study is that we only describe presence of MDSC in BM but not provide *in vivo* evidence that they indeed modulate intestinal immune responses. The first important step for BM-MDSC to have functional impact in the intestine is that they have to survive gastric and intestinal passage. To our knowledge, no data about survival of BM-leukocytes in human digestion models exists so far. However, some animal studies demonstrated *in vivo* an active BM-leukocyte transfer through the intestinal mucosa (37–40). After *in vitro* digestion with gastric and pancreatic enzymes, we still could detect GR-MDSC in BM. However, absolute percentages of BM-MDSC of total leukocytes were lower than prior to digestion as were expression levels of CD66b. From enzymatic tissue digestion it is known that expression of surface markers is often impaired (41). Beyond that, in previous experiments, we observed several times that surface phenotype of GR-MDSC may change under certain culture conditions and in particular expression of CD66b can disappear [discussed in Ref. (17)], so that we suppose that after mimicking gastric and intestinal digestion CD66b is shedded, leading to the lower percentages of BM-MDSC. Although our results point toward an impact of BM-MDSC on intestinal immune regulation, it still remains speculative that BM-MDSC indeed may have influence on inflammation control and pathogenesis of diseases like NEC. Further it remains elusive, whether they exert their suppressive activity directly in the intestine or if they cross the intestinal mucosa and reach the circulation and/or peripheral lymphoid organs. These questions will be addressed in continuing *in vivo* studies.

Next, we found that BM-MDSC exhibit an altered phenotype compared to blood GR-MDSC, with upregulation of chemokine receptor CXCR4 and coinhibitory molecules PD-L1 and PD-L2. As discussed previously (16), CXCR4 has been described as an MDSC activation marker in cancer (42). We have shown that CXCR4 was upregulated on placental MDSC and that blockade of CXCR4 reduced their suppressive activity (16). Similar to CXCR4, expression of PD-L1 on MDSC seems to be relevant for their suppressive activity (43, 44), while to our knowledge PD-L2 expression on MDSC has not yet been described. Thus, upregulation of CXCR4, PD-L1, and PD-L2 on BM-MDSC points toward an activated state that is in line with our finding of an increased suppressive activity of these cells. Interestingly, it has been shown that high CXCL12 levels—the ligand for CXCR4—in BM were associated with reduced risk for HIV-transmission to breastfed infants (45). The role of BM-MDSC and their regulatory functions for breastmilk-transmitted infections like HIV may be subject of further studies.

Concerning MDSC effector mechanisms we found an increased expression of iNOS and a decreased production of ROS in BM-MDSC compared to GR-MDSC from peripheral blood of pregnant women, while expression of ArgI and IDO were not different. This expression pattern clearly differs from enzyme expression of GR-MDSC during pregnancy, where we found an upregulation of ROS production by placental GR-MDSC (16). Production of NO by iNOS was shown to decrease expression of MHCII molecules on macrophages and thereby inhibit T-cell activation (46). NO-production by cord blood MDSC led to induction of Tregs (18). Both mechanisms may play a role in regulating intestinal inflammation through BM-MDSC. The functional role of increased iNOS expression by BM-MDSC will be further evaluated. On the whole, BM-MDSC express a distinct pattern of surface- and effector-molecules reflecting BM as an immunological niche with specialized immune effector cells.

Furthermore, we showed that BM-MDSC inhibit TLR4-expression on monocytes. TLR4 has been shown to play a critical role in NEC pathogenesis. TLR4-expression in intestinal mucosa was increased during NEC and lack of TLR4 in mice protected against NEC (26). Interestingly, the NEC-protective effect of BM has been attributed to reduced TLR4 expression in intestinal epithelium (27). Given our present findings, it may be hypothesized that MDSC in BM protect against NEC by downregulating TLR4 on monocytes.

Finally, we aimed to identify factors responsible for the accumulation of MDSC in BM. Studies in mice revealed that the sex hormones estradiol and progesterone lead to an induction of MDSC during pregnancy and cancer (35, 47, 48). Immune-modulatory properties have also been described for the lactotrophic hormones prolactin and oxytocin (49–51). However, we found no effect of oxytocin or prolactin on MDSC induction. Thus, the mechanism of MDSC-accumulation in BM yet remains unclear. Other potential mediators for MDSC-accumulation in BM are the Th2 cytokines IL-4 and IL-13, for which an overexpression in the lactating mamma has been described (52) and which are both implicated in the activation of MDSC (30, 53, 54). Accordingly, we found higher expression of IL4Rα in BM-MDSC than in GR-MDSC from peripheral blood of pregnant women; however, differences were not statistically significant. Examining the interactions between mammary epithelial cells and MDSC might give more insights in activation mechanisms of MDSC during lactation.

In conclusion, we describe for the first time GR-MDSC in BM, showing an activated phenotype and modulating TLR-expression on monocytes *in vitro*. These results provide evidence that BM-MDSC may play a role in regulating intestinal immune responses in neonates. Further studies are now needed to prove their functional role *in vivo*. Potentially, BM-MDSC could be a promising target for modulating neonatal inflammatory response locally in the gut, supporting establishment of the gut microbiome and potentially protecting neonates from NBI or NEC.

## Ethics Statement

This study was approved by the Ethics committee of the Medical Faculty of Tuebingen’s University (No. 682/2016BO1). All subjects gave written informed consent in accordance with the Declaration of Helsinki.

## Author Contributions

NK and CG conceptualized and designed the study; CS, JS, BS, RG, and AM collected samples and data; NK, CS, GB, and BS performed experiments; NK, CS, and CG analyzed data; NK drafted the initial manuscript; NK, CS, RG, CP, and CG reviewed and revised the manuscript. All authors approved the final manuscript as submitted and agreed to be accountable for all aspects of the work.

## Conflict of Interest Statement

The authors declare that the research was conducted in the absence of any commercial or financial relationships that could be construed as a potential conflict of interest.

## Abbreviations

ArgI, arginase I; BM, breast milk; CBMC, cord blood mononuclear cells; DHR, dihydrorhodamine; GR-MDSC, granulocytic MDSC; IDO, indolamin-2,3-dioxygenase; iNOS, inducible NO synthase; MDSC, myeloid derived suppressor cell; MO-MDSC, monocytic MDSC; NBI, nosocomial bacterial infection; NEC, necrotizing enterocolitis; PBMC, peripheral blood mononuclear cells; ROS, reactive oxygen species; TLR, toll like receptor; VLBW, very low birth weight.
