# Supplementary material for: Granulocytic Myeloid-Derived Suppressor Cells (GR-MDSC) in Breast Milk (BM); GR-MDSC Accumulate in Human BM and Modulate T-Cell and Monocyte Function
Source: Front Immunol. 2018 May 17;9:1098. doi: 10.3389/fimmu.2018.01098 (PMC5966528; doi:10.3389/fimmu.2018.01098)
Supplement: Supplementary file 1 [file image_1.PDF]

Supplementary Figure 1

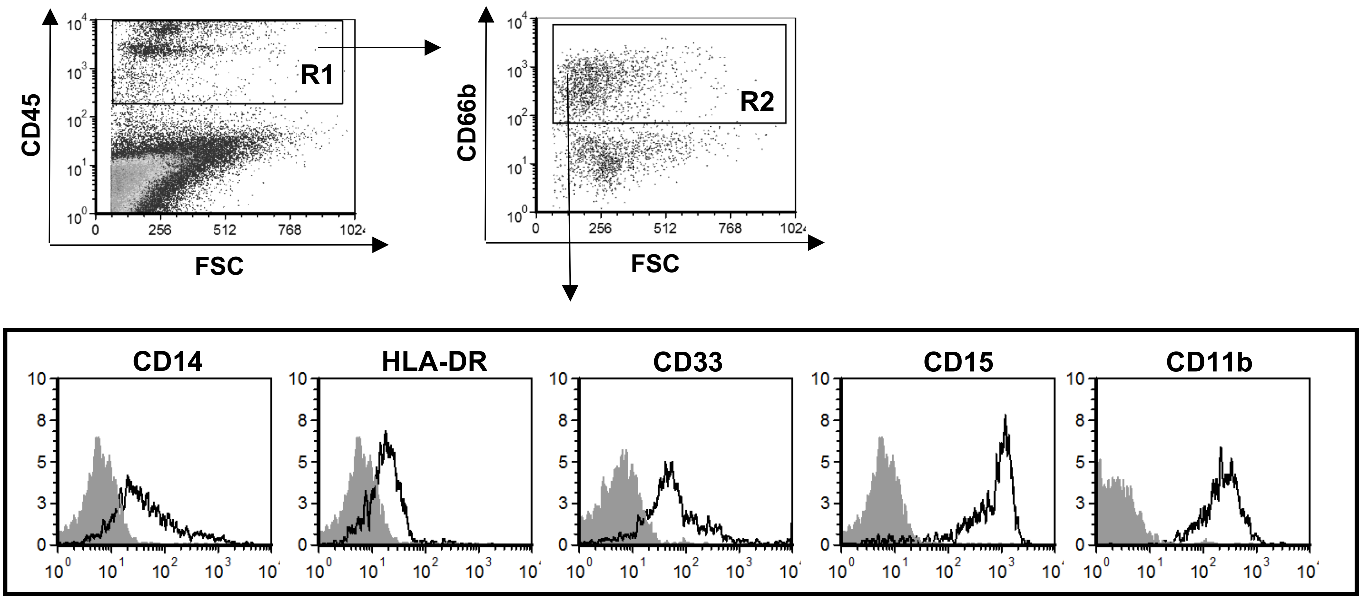

Supplementary Figure 1: Expression of surface markers on BM-MDSC

Milk cells were isolated from breast milk and analysed by flow cytometry. Densitiy plots for forward scatter (FSC) versus CD45 and forward scatter (FSC) versus CD66b showing gating strategy of BM-MDSC. R1 shows brest milk comprising leucocytes. Gate R2 shows the population of BM-MDSC. Histogram plots showing expression of surface markers CD14, HLA-DR, CD33, CD15 and CD11b on BM-MDSC from gate R2.

Supplementary Figure 2

A

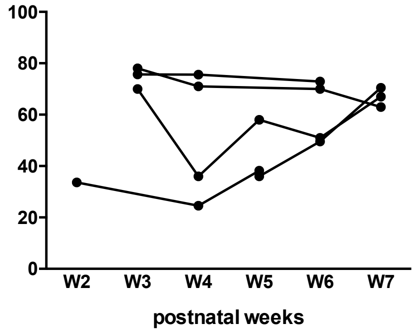

Supplementary Figure 2: Development of GR-MDSC count in mother´s milk of preterm infants during puerperal periode

Milk cells were isolated from mother´s milk of preterm infants and analysed by flow cytometry. Progression chart shows percentages of GR-MDSC in mother´s milk collected in weekly intervals between the 2nd postnatal week (W2) to the 7th postnatal week (W7).

Supplementary Figure 3

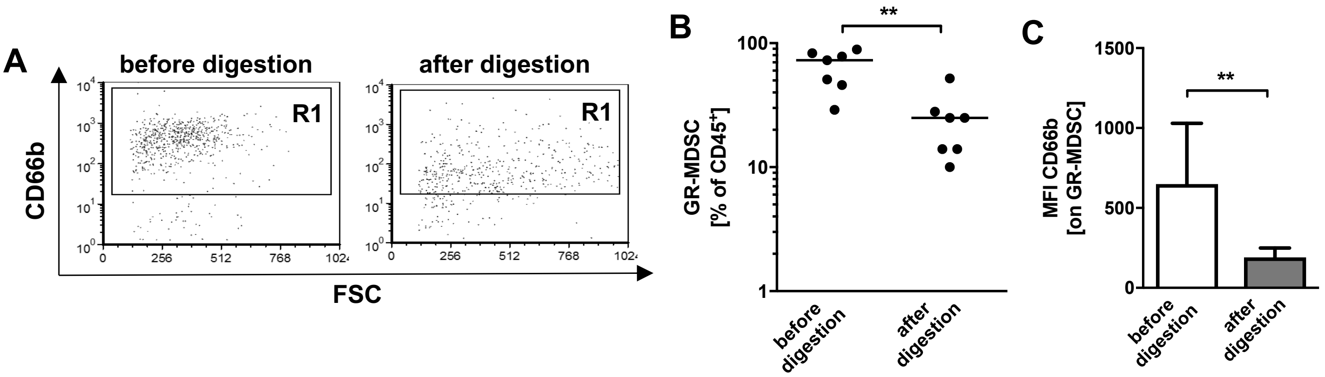

Supplementary Figure 3: BM-MDSC after *in vitro* digestion

Breast milk was incubated with gastric and pancreatic enzymes according to a previous established protocol by Klitgaard et al.. Afterwards, milk cells were isolated from breast milk and analysed by flow cytometry. Non-digested breast milk served as control. (A) Densitiy plots for forward scatter (FSC) versus CD66b showing gating strategy of BM-MDSC before and after digestion. R1 shows breast milk comprising BM-MDSC. (B) Scatter diagram shows percentages of BM-MDSC from total leukocytes before and after enzymatic digestion. Plots represent seven independent experiments. Each symbol represents an individual sample and the median is indicated. $^{**}p < 0.01$; Wilcoxon matched pairs signed rank test. (C) Bar graph shows mean fluorescence intensity (MFI) of CD66b on BM-MDSC. Plots represent seven independent experiments. Mean and standard deviation are indicated. $^{**}p < 0.01$; Wilcoxon matched pairs signed rank test.

Supplementary Figure 4

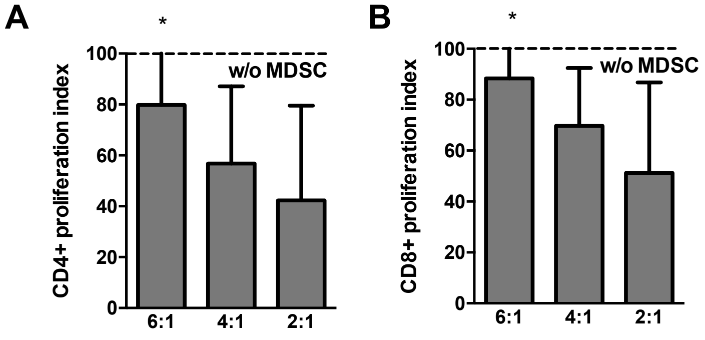

Supplementary Figure 4: Inhibition of T-cell proliferation in cord blood by MM-MDSC

GR-MDSC were enriched from mother´s milk and added to CFSE-stained and IL-2/OKT3-stimulated CBMC isolated from a healthy term born term neonates. After four days, proliferation of $CD4^+$ and $CD8^+$ T-cells was assessed by CFSE dye dilution. Proliferation index was determined as ratio of T-cell proliferation with and without addition of GR-MDSC and T-cell-proliferation in different proportions. (A, B) The inhibitory effect of GR-MDSC on proliferation of $CD4^+$ (A) and $CD8^+$ T-cells (B) was assessed. Dashed line shows proliferation of target CBMC without addition of GR-MDSC. Inhibition of T-cell proliferation by GR-MDSC isolated from mother´s milk was measured at the indicated ratios by CFSE dye dilution. Bars show mean and standard deviation of 7 samples pooled from 7 independent experiments. *$p<0.05$ compared with target cells alone; Wilcoxon matched-pairs signed-rank test.
